# Mineralocorticoid Receptor Antagonists—Use in Chronic Kidney Disease

**DOI:** 10.3390/ijms22189995

**Published:** 2021-09-16

**Authors:** Wiktoria Baran, Julia Krzemińska, Magdalena Szlagor, Magdalena Wronka, Ewelina Młynarska, Beata Franczyk, Jacek Rysz

**Affiliations:** Department of Nephrology, Hypertension and Family Medicine, Medical University of Lodz, ul. Żeromskiego 113, 90-549 Łódź, Poland; wikaa17@gmail.com (W.B.); julia.krzeminska1@gmail.com (J.K.); szlagor.magdalena@gmail.com (M.S.); wronkam96@gmail.com (M.W.); bfranczyk-skora@wp.pl (B.F.); jacek.rysz@umed.lodz.pl (J.R.)

**Keywords:** mineralocorticoid receptors antagonist (MRA), chronic kidney disease (CKD), diabetic nephropathy (DN), patiromer, albuminuria

## Abstract

Mineralocorticoid receptor antagonists (MRA) are drugs with a potentially broad spectrum of action. They have been reported to have healing effects in many diseases, such as chronic heart failure, hypertension, or nephrotic syndrome. Numerous studies suggest that mineralocorticoid receptor activation is pathogenic and a progression factor of chronic kidney disease (CKD); however, results of studies on the use of MRA in the treatment of CKD are inconclusive. Current guidelines recommend against the use of MRA in patients with advanced CKD. Although, there is growing interest on their use in this population due to treatment benefits. In this review, we summarize studies which were purposed to evaluate the impact of MRA therapy on CKD patients. Despite many benefits of this treatment e.g., reducing cardiovascular mortality or alleviating proteinuria, steroidal MRA (such as spironolactone or eplerenone) have a low safety profile. They often lead to hyperkalemia complications which are dangerous in patients with CKD, and diabetic nephropathy, especially in hemodialysis patients. Studies on recently developed nonsteroidal MRA showed that they have fewer side effects. In our review, we discuss steroidal and nonsteroidal MRA treatment effects on the estimated glomerular filtration rate (eGFR), proteinuria, the cardiovascular system, and hyperkalemia in CKD patients. We present new content and recent publications in this field.

## 1. Introduction

The renin–angiotensin system (RAS) stimulates, among others, the secretion of aldosterone hormone, which participates in the control of blood pressure (BP), the volume of extracellular fluid or potassium serum levels. Activation of the mineralocorticoid receptor (MR) stimulates not only salivary and sweat gland ducts, aldosterone-sensitive distal nephrons and the colon, but also has multiple effects on cardiomyocytes, fibroblasts, podocytes, endothelial cells, and many others [1], which lead to cardiac hypertrophy, fibrosis and proinflammatory effects [1,2].

Mineralocorticoid receptor antagonists (MRA) are an essential element in the treatment of chronic kidney disease (CKD) due to their anti-inflammatory and antifibrotic effects [2]. The first invented MRA was spironolactone, which has nonselective actions and affects androgen and progesterone receptors [3]. Nowadays there are also two other groups of MRA available, the more selective eplerenone [2,4] and the novel, nonsteroidal, selective MRA—finerenone, esaxerenone, apararenone [2,5]. Among all MRA, nonsteroidal new-generation MRA appear to have better anti-inflammatory and antifibrotic effects; moreover, they cause minimal hyperkalemia and a lesser decrease in the estimated glomerular filtration rate (eGFR) during therapy [2,3]. This treatment seems to be beneficial especially in patients with type 2 diabetes [2]. Numerous studies have proven the beneficial effects with ejection fraction (EF) of MRA in patients with heart failure (HF) [6]. More and more data show a protective action on renal function by lowering albuminuria in patients with chronic kidney disease, especially in patients with type 2 diabetes, declining the progression of CKD and reducing cardiovascular (CV) outcomes, with however, an increased risk of hyperkalemia [1,7].

CKD is defined as “abnormalities in kidney structure or function which are present for more than 3 months and have health implications” [8]. According to the KDIGO 2012 guideline, CKD is classified by the cause, glomerular filtration rate (GFR) category, and albuminuria category [8]. CKD is a substantial medical problem in the world and its occurrence is constantly increasing [7]. Furthermore, it is a significant risk factor for CV and all-cause mortality [9]. Conditions that lead to kidney dysfunction are obesity or type 2 diabetes; moreover, in Japan, the last one is a primary cause of end-stage renal disease (ESRD) [10,11].

Proteinuria is a noted risk factor for the progression of CKD [10]. RAS blockers are efficient and commonly used as renoprotective and antialbuminuric drugs; however, they are not enough to reduce proteinuria and slow down the progression of renal failure [11]. This treatment reduces the rate of decline of CKD from 10–12 to 2–3 mL/min/1.73 m^2^ per year; nevertheless, a normal decline should be less than 1 mL/min/1.73 m^2^ per year. Angiotensin-converting enzyme inhibitors (ACEI) and angiotensin II receptor blockers (ARB) do not affect aldosterone, and its levels in plasma increase despite the treatment. MRA therapy induces an appreciable reduction in proteinuria, which results in a delay of the progression of renal failure and may contribute to a slower rate of decline in GFR [12].

Despite their widespread use, aldosterone blockers have their limitations. The most common is the increased risk of hyperkalemia, defined as serum potassium >5.5 mmol/L. It may be exacerbated when ACEI are used concomitantly in the elderly, diabetic, or dehydrated patients. However, the patients most at risk of hyperkalemia are those on hemodialysis with end-stage HF [12]. Currently, more and more is said about potassium binders that stand out from other hyperkalemia treatment strategies. One of them is a new ion exchange resin—patiromer, which can be used in people with severe renal impairment and heart or liver failure [13]. It represents a significant pharmacologic advancement in the treatment of hyperkalemia, exhibiting significant reductions in serum potassium in patients with CKD [14].

In this review, we present and summarize the available data relating to the safety and efficacy of MRA therapy in patients with CKD. Due to the fact that MRA are divided into steroidal and nonsteroidal chemicals, we discuss their benefits and drawbacks focusing on eGFR, proteinuria, the CV system, and hyperkalemia.

## 2. Spironolactone or Eplerenone? Use in Therapy in Patients with CKD

Yang et al. [15] in “The Long-term Effects of Spironolactone on Kidney Function and Hyperkalemia-Associated Hospitalization in Patients with Chronic Kidney Disease” conducted a study with over 3 years of follow-up. Participants of the study were 693 spironolactone users and 1386 patients who were not treated with spironolactone. All the patients were in stage 3–4 CKD. In the nonselective MRA users’ group versus spironolactone, the nonuser’s prevalence of end-stage renal disease (ESRD) was lower (12.7% versus 19.19% *p* < 0.001). Nonetheless, patients on spironolactone treatment had an increased prevalence of hyperkalemia hospitalization, 17.75% vs. 6.64% of nonusers. Only in the subgroup of patients with diabetes was a significant interaction apparent, with ESRD associated with spironolactone [15]. However, the study of Wang et al. [16] does not confirm that spironolactone has a protective effect on the development of ESRD. In the study, differences in renal function, eGFR, potassium level, aldosterone, or BP were not noticeable. Attention should be paid to the urine protein level, noted at every 4 weeks of treatment for 16 weeks, which was significantly decreased in the spironolactone users’ group [16]. In addition, an increased potassium level during MRA therapy is a frequent adverse effect. Momeni et al. [17] in their study estimated the connection of spironolactone with hydrochlorothiazide in patients with diabetic nephropathy (DN). It turned out that not only was 24-h urine protein decreased (*p* < 0.0010), reducing the development of advanced DN, but also adverse effects in the form of increased potassium levels in patients with hydrochlorothiazide treatment were not noticed [17].

Scientists are not only concerned with spironolactone treatment in CKD, but many also study the effect of eplerenone treatment. In the study, Eplerenone in Mild Patients Hospitalization and Survival Study in Heart Failure (EMPHASIS-HF), the effect of different doses of eplerenone versus placebo stratified by renal function were examined. EMPHASIS-HF was a randomized, double-blind trial with a median follow-up duration of 21 months. The trial included 2737 patients with NYHA II and a left ventricular ejection fraction (LVEF) ≤35%. The primary outcome was hospitalization for HF or death caused by cardiovascular disease (CVD). Different doses of eplerenone were related to renal function. In the EMPHASIS-HF trial the high dose of eplerenone was as effective as the low dose in the assigned groups [18]. However, the study of a subgroup with a higher risk of hyperkalemia showed that despite an increased risk of potassium 5.5–6 mmol/L, and hospitalization for an increased serum potassium level or discontinuation of the study medication, eplerenone was effective in reducing the primary outcomes in all subgroups [19]. What is more, the trial conducted by Epstein et al. [20] showed that eplerenone treatment significantly reduced albuminuria and decreased SBP. However, there was no significant difference in the median eGFR during the follow-up in each group. In the treatment of albuminuria, a lower dose of eplerenone should be preferable to a higher one, because of the lower risk for hyperkalemia. Co-administration of eplerenone with ACEI significantly reduces albuminuria in patients with diabetes and does not significantly increase hyperkalemia [20].

## 3. Novel Selective Nonsteroidal MRA Therapy in Patients with CKD

Steroidal MRA have plenty of limitations, which is why scientists try to find drugs with fewer side effects for clinical treatment by studying the impact of novel selective nonsteroidal MRA. So far, the best known is finerenone.

Short-term studies in patients with CKD and type 2 diabetes have shown that the use of finerenone reduces albuminuria [21]. Moreover, the study of Bakris et al. [22] clearly showed the effect of finerenone treatment on kidney outcomes. Patients who received finerenone had a lower risk of death from renal causes, and sustained a decrease of ≥40% in the eGFR or kidney failure. However, even finerenone treatment may lead to hyperkalemia [22]. In addition, the phase 2B study, MinerAlocorticoid Receptor antagonist Tolerability Study-Diabetic Nephropathy (ARTS-DN), compared the effectiveness of nonsteroidal MRA—finerenone in different doses to placebo in DN. In 90 days of follow-up, a decrease in the urinary albumin-creatinine ratio (UACR) that correlated with finerenone doses was noted. The study did not confirm differences in the secondary outcome of a 30% eGFR decrease or; moreover, in the incidence of adverse events between the placebo and finerenone groups. Finerenone treatment may increase serum potassium levels, but in the placebo and finerenone 10 mg group there were no discontinuations of therapy due to hyperkaliemia [23].

However, scientists have evaluated the efficacy and safety of the other novel MRA which are esaxerenone and apararenone. In a study which aimed to evaluate the effects of esaxerenone treatment, the intervention group was compared to the placebo group. UACR remission, as well as a reduction of the progression of albuminuria, were achieved. Unfortunately, and likewise in most other studies, there was a risk of hyperkalemia [24]. Similar results were achieved by Wada et al. [25] The study compared the efficacy and safety of apararenone treatment vs. placebo in patients with stage 2 DN. During the follow-up, UACR decreased. Hyperkalemia and a decreasing eGFR were observed, but with no clinical significance [25].

## 4. Safety and Efficacy of MRA Therapy in Diabetic Kidney Disease

According to the American Diabetes Association’s guidelines, the definition of diabetic kidney disease (DKD) is based on clinical parameters, which include albuminuria and/or a decreased eGFR in patients suffering from diabetes with the exclusion of other causes of kidney damage [26]. DKD occurs in 20–40% of diabetic patients and is one of the most common complications of both types of diabetes [26,27]. Data concerning the use of MRA in clinical practice indicate that they are rarely used in patients with CKD (1.2%), however, it has been observed that MRA therapy is used more often in patients with DKD (1.8%) and with DKD and HF (6.6%) [28]. It was observed that patients with type 2 diabetes and CKD using MRA, compared to patients not taking spironolactone, were more likely to develop comorbidities. It was reported that a greater proportion of patients taking spironolactone compared to those not treated with MRA, experienced acute kidney injury (AKI) (51.1% versus 33.9%), hyperkalemia (29.9% versus 17.2%), and progression to a more advanced stage of kidney disease (higher grade, ESRD or renal replacement therapy (RRT)) within 1 year (29.9% versus 18.4%). Patients treated with spironolactone required hospitalization more often than those not taking MRA. Blankenburg et al. emphasized that DKD patients who took spironolactone had a greater burden of comorbidities than those who did not receive MRA therapy, hence the poorer treatment outcomes for patients treated with spironolactone [29]. Besides, Kato et al. indicated that in patients with type 2 diabetes and nephropathy, spironolactone could be recommended as a second line of therapy in the event of insufficient antihypertensive and antialbuminuric effects of RAS blockers [11].

## 5. The Effects of MRA Therapy on the Estimated Glomerular Filtration Rate

eGFR is a fundamental parameter of renal function [18]. There are randomized controlled trials that have investigated the effects of MRA therapy on eGFR [30]. Recent studies have evaluated the effects of classical steroidal MRA such as spironolactone, canrenone, and eplerenone, as well as new nonsteroidal MRA such as finerenone [12], esaxerenone [24], and apararenone [25]. The results showed that in the group of patients with stage two and three CKD and a high plasma aldosterone concentration, the use of MRA may be effective in preventing the progression of CKD [30].

As obesity is a risk factor for the development of CKD, proteinuria contributes to its progression [10], and DN is a leading cause of CKD [31], the effects of MRA therapy on eGFR have been studied concerning CKD-related disease entities. Data on the effect of MRA plus ACEI/ARB therapy in patients with type 2 diabetes are not conclusive [11,31]. Kato et al. reported that the addition of spironolactone to antihypertensive treatment with RAS blockers, in patients with type 2 diabetes mellitus, an eGFR >30 mL/min/1.73 m^2^ and persistent albuminuria, resulted in a decrease in eGFR (3.2 ± 9.7 mL/min/1.73 m^2^) [11]. The meta-analysis by Sun et al. [31] revealed that in patients with DN, ACEI/ARB therapy with MRA compared to ACEI/ARB monotherapy did not improve GFR. It is supposed that MRA plus ACEI/ARB treatment maintains the GFR relative stability in DN patients [31]. In Morales et al. [10] study, patients with proteinuria >1 g/24 h who were taking spironolactone and another renin–angiotensin–aldosterone system (RAAS) blocker, were divided into the obesity group (body mass index (BMI) ≥30 kg/m^2^) and the control group. Overall, there was a slight improvement in eGFR in the pretreatment period to the end of the follow-up. Improvement in renal function was noted in 62.5% of obese patients, compared to 59% in the nonobese group [10]. Moreover, the authors of the EMPHASIS-HF study suggest that in patients with CVD the dose of eplerenone should be adjusted according to renal function [18]. However, Alexandrou et al. reported that the use of MRA in monotherapy or on top of RAS blockade, compared with placebo, is associated with a decrease in the eGFR of −2.38 mL/min/1.73 m^2^ (95% CI −3.51 to −1.25) [12].

Research on nonsteroidal MRA has shown that in a group of patients with type 2 diabetes and microalbuminuria treated with RAS inhibitors, the addition of esaxerenone resulted in a reduction of eGFR by an average of 10%. In patients taking esaxerenone, the decline in eGFR was greatest during the first 24 weeks of treatment, then stabilized and was similar to those in the placebo group during the post-treatment follow-up period (Ito et al. [24]). Furthermore, Bakris et al. [22] proved that adding finerenone to the therapy of patients with CKD and type 2 diabetes treated with ACEI/ARB resulted in a less frequent decrease in eGFR (by at least 40% from baseline and by at least 57% from baseline) than in the placebo group.

In summary, the effects of MRA therapy on eGFR are inconclusive. However, it is noteworthy that the studies conducted differ with respect to comorbidities and MRA used. In four of the seven studies, eGFR decreased after MRA therapy [11,12,22,24], in one there was a nonsignificant improvement [10], while in the other two eGFR did not improve [31] and remained at an equal level [30]. The results are presented in Table 1.

## 6. Proteinuria Response during MRA Therapy

In patients with kidney dysfunction, higher values of proteinuria may contribute to the progression of renal disease. However, the use of any treatment that reduces proteinuria may cause a satisfactory and renoprotective effect [10].

A meta-analysis undertaken by Sun et al. [31] has estimated the potential benefits and side effects of the addition of an MRA to the standard treatment of DN, which consists of an ACEI and/or an ARB. It was suggested that the progression of DN may be accelerated by an increased urinary albumin excretion (UAE) and UACR levels and that reducing these parameters may favorably affect renal outcomes. This meta-analysis has shown that the combination of MRA with ACEI/ARB causes appreciable improvement of UAE and UACR levels in comparison to their values after ACEI/ARB treatment [31]. A prospective, randomized study undertaken by Kato et al. [11] was another trial related to patients with type 2 diabetes. The aim of the study was the evaluation of the efficacy and protection of spironolactone in patients who were already treated with ACEI/ARB. The primary endpoint was the reduction in albuminuria assessed with UACR after 8 weeks of treatment. A substantial reduction in albuminuria was noticed in patients who received the additive spironolactone treatment in comparison to the nonspironolactone group. This study also suggests that spironolactone reduces albuminuria independently of systemic hemodynamic alterations [11]. Likewise, a randomized clinical trial conducted by Esteghamati et al. [32] included patients with proteinuria and diabetes, already treated with losartan and enalapril. After 18 months of the study, it was shown that the spironolactone-ARB combination also reduced UAE by 46% after 3 months, 72% after 12 months, and 59% after 18 months. The discussed study also confirmed that the use of therapy consisting of spironolactone with ACEI or ARB may provide a better antiproteinuric effect than the use of one medication [32].

A randomized controlled clinical trial performed by Sadayoshi Ito and colleagues also focused on patients with type 2 diabetes. During this 52-week study, it was observed that the addition of esaxerenone to RAS inhibitors led to urinary albumin-to-creatinine remission in 22% of patients versus 4% of patients in the placebo group. Moreover, a significant improvement in the percentage change in UACR was noticed from the beginning to the end of the therapy in the esaxerenone group (−58%) rather than the placebo group (8%). The present study demonstrated that the combined therapy led to an increased percentage of patients with a 30% reduction in UACR (69%) [24]. Similar conclusions can be drawn from the randomized, controlled study undertaken by Wada et al. [25]. During the trial, 73 patients were in the placebo group and 73, 74 and 73 patients received apararenone treatment in doses of 2.5 mg/day, 5 mg/day and 10 mg/day, respectively. It was confirmed that apararenone treatment at the dose of 2.5–10 mg/day for 24 weeks caused a greater reduction of UACR than placebo. Moreover, a higher reduction in UACR was noted for the 5 mg and 10 mg apararenone groups than for the 2.5 mg group. From the beginning, the percentage change in the UACR in the 2.5 mg apararenone group was around 40% and in the 5 mg and 10 mg groups was around 50% after 24 weeks of study [25].

A separate meta-analysis was conducted by Gemma Currie and colleagues who analyzed 19 trials investigating the renoprotective effects of MRA therapy in patients with CKD. This study demonstrated that a therapy consisting of MRA with ACEI and/or ARB did not cause a significant change in the albumin:creatinine ratio, but a substantial difference in the 24-h UAE (−332.9 mg/24 h) was noticed. It was likewise observed that this therapy appreciably changed protein:creatinine ratio values (−0.91 g/g creatinine) compared with the ACEI and/or ARB treatment. This meta-analysis demonstrated that the addition of MRA to ACEI and/or ARB led to a 38.7% reduction of urine protein/albumin excretion [33]. Similar conclusions were observed in a clinical trial performed by Morales et al. [10] which aimed to investigate the renoprotective effects of a combination of RAS blockers with MRA therapy in patients with proteinuric nephropathy and obesity. During the study, a substantial reduction in proteinuria (68.2%) was noticed in the obesity and control groups. A 46.3% reduction in proteinuria was observed from baseline values in the obesity group during spironolactone treatment. These findings showed that in the obese group MRA treatment induced a notable and efficient reduction in proteinuria, but the nonobese group demonstrated better results [10]. A comparison of studies showing the effect of combination therapy of MRA with ACEI/ARB on proteinuria is presented in Table 2 [10,11,24,25,31,32].

## 7. Biomarkers Predicting the Albuminuria Response

Nowadays there are new methods that increase diagnostic opportunities and identify patients with a higher risk of progression to proteinuria, especially patients with type 1 and 2 diabetes [34]. In order to enable the prediction of the albuminuria response to spironolactone therapy, and thus to also better adapt this therapy in patients with type 2 diabetes, the identification of relevant biomarkers was undertaken [34,35,36].

Skander Mulder and colleagues [35] have tried to identify a set of urinary metabolites which would predict the response of albuminuria to the spironolactone treatment in type 2 diabetes. Using bioinformatics analysis, they have distinguished a set of 18 metabolites associated with a molecular model of DN that could be influenced by spironolactone therapy. This study has shown that the mentioned urinary metabolites reflected by the fibrosis process may be a useful instrument to personalize therapy in patients with type 2 diabetes and predict the spironolactone treatment response [35].

A proteomic classifier based on 273 urinary peptides (CKD273) was developed and validated. Lindhardt et al. [34] have demonstrated that the measurement of 273 specific urine peptides (CKD273), the higher levels of which are related to advanced DN and renal fibrosis, enables the identification of patients with a better response to the MRA spironolactone treatment. This implies that an alteration of proteins in the urine, causing a higher CKD273 score, makes the individual more susceptible to treatment. By means of urinary proteomics, they demonstrated the highest reduction in UACR in patients with type 2 diabetes with a higher CKD273 score after 16 weeks of spironolactone treatment. This correlation was not noticed in the group of patients taking a placebo. These results suggest that urinary proteomics may be a useful instrument to select individuals at a greater risk of progression in DN and those who will benefit from MRA treatment, as it is a predictor of the occurrence of an albuminuria-lowering response to spironolactone treatment [34]. Similar subject matter was discussed in PRIORITY, the study by Tofte et al. [36]. Patients with type 2 diabetes, a normal UAE, and preserved renal function were classified as high risk (CKD273 classifier score >0.154) or low risk (≤0.154) based on a urinary proteomic risk classifier—CKD273. Compared with low-risk participants, a larger proportion of high-risk patients experienced microalbuminuria (28% versus 9%), impaired renal function (eGFR <60 mL/min/1.73 m^2^) (26% versus 8%) and a decrease in eGFR by 30% from baseline (19% versus 4%). Nevertheless, no significant difference was observed in the high-risk patient group—the development of microalbuminuria was observed in 33% of patients in the placebo group and 25% of patients taking spironolactone. The study proved that the patients, who were classified as high risk based on a urinary proteomic risk classifier—CKD273, had an increased risk of progression to microalbuminuria and decreased renal function. However, treatment with spironolactone has not been shown to prevent the progression of microalbuminuria for these patients [36].

## 8. Effect on the Cardiovascular System in Patients with Renal Diseases

Presumably, MRA may reduce the risk of CV mortality in patients who require dialysis. Patients with ESRD receive dialysis. These patients are burdened with a 20% increased mortality rate and the leading cause of death is CV disease. HF may be developed with the preserved EF or without it; however, this condition may develop by a different mechanism, unlike non-dialysis-dependent patients. Given the available data, the use of MRA therapy in dialysis patients is uncertain due to the unclear benefits and risks [37].

Lin et al. [38] have conducted a multicentric, randomized, and placebo-controlled study aiming to evaluate the long-term outcomes and side effects of spironolactone, on cardiocerebrovascular (CCV) protection, and other clinical parameters among non-HF dialysis patients with ESRD. Patients were assigned to the spironolactone or control group. During this trial some CCV-related parameters such as LVEF, left ventricular mass index (LVMI), heart rate variability, and the number of antihypertensive drugs were analyzed in these two groups, and in the spironolactone group a significant improvement was noticed. The primary endpoint was represented by death from sudden cardiac death, death from CCV events, and aborted cardiac arrest. Of the patients, 7.2% from the spironolactone group and 18.0% from the control group reached the primary endpoint. Furthermore, 4.0% of patients from the spironolactone group and 11.7% of patients from the control group died from CCV events. The present study demonstrated that a low dose of spironolactone therapy can efficiently reduce CCV events—morbidity and mortality; furthermore, it also reduces left ventricular size. The use of spironolactone in dialysis patients with ESRD may successfully reduce the CCV mortality by its CCV protective effect [38]. Similar conclusions can be drawn from a randomized, double-blind trial undertaken by Feniman-De-Stefano and colleagues [39] who estimated the efficacy and protection of spironolactone treatment in reducing the left ventricular mass in patients who require hemodialysis. These patients received 12.5 mg of spironolactone, which was titrated in the next week, to a dose of 25 mg or placebo. In the spironolactone group, there was a LVMI reduction from 77 ± 14.6 g/m to 69 ± 10.5 g/m, *p* < 0.04; however, in the placebo group an increase from 71 ± 14.2 g/m to 74 ± 17.4 g/m was detected. From the ambulatory blood pressure monitoring (ABPM), a parallel decrease of awake ABPM pulse pressure, and pulse pressure in 24-h was noticed in both groups after therapy. The results have shown that LVH may be a surrogate endpoint in hemodialysis patients, predicting a better survival during spironolactone therapy. Moreover, spironolactone treatment seems to be a safe and efficient therapy in the regression of left ventricular hypertrophy (LVH) [39]. In the FIDELIO-DKD trial, the effect of finerenone added to RAS blockade on the kidney and CV system was estimated. The discussed trial included individuals with type 2 diabetes, DN, and a UACR of 30–5000 mg/g, and patients were assigned to receive finerenone or placebo. The clinical trial has shown that finerenone reduces the risk of the composite CV events among individuals with type 2 diabetes and CKD, independently from a history of CV disease. The results suggest that finerenone therapy may be used for primary as well as secondary CV prevention in these patients [40]. Likewise, the Finerenone in Reducing CV Mortality and Morbidity in Diabetic Kidney Disease (FIGARO-DKD) trial, is a study that aims to compare finerenone to placebo in reducing clinically important CV and renal outcomes in patients with CKD and type 2 diabetes [41].

Similar but inconclusive findings were observed by Quach et al. [37] who made a systematic review and meta-analysis of MRA in dialysis patients. They suggest that MRA may presumably be a profitable therapy, reducing CV mortality in patients who require dialysis; however, data are deficient to determine clearly whether MRA are beneficial treatments. In dialysis patients after MRA, a 66% relative risk (RR) of reduction for major CV events was noted, but only some of them had HF. It was emphasized that MRA is a promising therapy, but extensive research is required to define the benefit–risk ratio [37]. Likewise, a meta-analysis conducted by Currie et al. [33] has demonstrated a substantial difference in BP at the last visit in patients with a 1–5 CKD stage after MRA treatment. Addition of MRA to ACEI and/or ARB therapy caused a reduction in SBP (−5.7 mmHg) as well as DBP (−1.7 mmHg) compared to ACEI and/or ARB alone at the end of the study. It was observed that the combination of ACEI and/or ARB with MRA significantly reduces BP as well as proteinuria which was reduced by 40%; therefore, we can expect greater benefits in CV disease prevention. However, despite potential CV benefits, conclusions on long-term protection and efficacy of MRA and/or ARB therapy cannot be drawn [33]. Nevertheless, a randomized and placebo-controlled study, MiREnDa, revealed no appreciable difference in LVMI (−2.86 ± 11.87 vs. 0.41 ± 10.84 g/m2), 24-h ambulatory BP, LVEF, and 6-min walk test distance between the spironolactone group and control group. Mentioned parameters did not change despite the use of 50 mg spironolactone compared with placebo [42,43]. Table 3. Presents the objectives and discussed the conclusions of the articles described above [37,38,39,40,43].

## 9. Hyperkalemia

Hyperkalemia is one of the most common and at the same time one of the most dangerous complications of the use of aldosterone antagonists. Its most serious consequences include ventricular arrhythmias or cardiac arrest [12]. The question arises whether these drugs are safe for patients diagnosed with CKD, which in most cases is associated with elevated potassium levels resulting from dysfunction of the renin–angiotensin–aldosterone system [44].

A study in a group of 17 dialysis patients showed that the level of potassium concentration did not differ significantly between the study group receiving spironolactone and the control group receiving placebo. However, it should be emphasized that the participants were subjected to continuous pharmacological monitoring. Besides, one of the exclusion criteria was, among others, hyperkalemia [39]. In turn, Michael Walsh in his work, noted that hyperkalemia appeared more often in patients who took eplerenone than in the placebo group. However, he emphasized that it can be safely used in patients without a previous history of hyperkalemia, provided that potassium levels are monitored—especially during the first 4 weeks of therapy [45].

The meta-analysis undertaken by Alexandrou et al. [12] analyzed studies comparing MRA to a placebo/active control. Its results showed that MRA increased kalemia by 0.22 mEq/L. Spironolactone raised potassium by 0.28 mEq/L compared with any type of treatment, whereas for eplerenone it was by 0.13 mEq/L. The authors also emphasized that we should not be afraid of the risk of hyperkalemia because of the potential benefits of taking MRA [12].

Another study showed that a mean increase in potassium (0.19 mmol/L) at the end of MRA treatment did not correlate with the risk of clinical complications of hyperkalemia. In addition, it has been demonstrated that diabetic patients with CKD are not at greater risk of increasing potassium levels compared to nondiabetic patients [33].

In the study by Lin et al. [38] no significant differences in the concentration of potassium were noticed between people who underwent hemodialysis and people who underwent peritoneal dialysis, although it has been shown that spironolactone poses a lower risk of hyperkalemia in dialysis patients compared to non-dialysis patients. This is because potassium metabolism occurs slightly in the kidney, but mainly through the excretion by dialysis [38]. It is also possible that dialysis-dependent patients may tolerate hyperkalemia better than non-dialysis-dependent patients due to frequent monitoring [37].

Novel nonsteroidal MRA appear to demonstrate a better benefit–risk ratio than steroidal MRA, where risk is measured as the propensity for hyperkalemia. The results obtained by Kolkhof et al. [5] show that finerenone poses a lesser risk of hyperkalemia due to the differential distribution of the drug in the heart and kidney [5]. In the case of esaxerenone, it has been shown that the profile of serum potassium levels remains stable in high-risk patient groups—with moderate renal dysfunction or diabetes with albuminuria. However, in patients with moderate renal impairment and type 2 diabetes and albuminuria, potassium levels increase following the initiation of treatment and after each dose escalation [46].

The results of above mentioned studies are presented in Table 4, although most of them showed an increase in serum potassium after the introduction of MRA, it could be controlled with a low-potassium diet, cation exchange resins, or low-dose thiazide diuretics [10]. It should also be emphasized that patients prescribed MRA require regular monitoring of potassium levels throughout the treatment period.

## 10. Minimizing the Risk of Hyperkalemia

Hyperkalemia is one of the reasons for MRA withdrawal. This decision reduces the risk of life-threatening consequences of high serum K^+^, but on the other hand, it causes the loss of the full benefits of treatment [47]. Thus, it seems that the prevention of hyperkalemia is an ideal solution that not only increases patient safety but also improves the therapeutic efficacy of MRA [48].

One of the primary strategies to reduce the risk of hyperkalemia is a lower dose of MRA. In the study by Rossing et al. [49], patients received spironolactone at a dose of 25 mg/day. Only one of the 21 subjects was excluded from the study due to excessively high K^+^ levels [49]. In contrast, when the dose of spironolactone reached 50 mg, the risk of hyperkalemia was significantly higher [50]; however, this dose increased the risk of moderate but not severe hyperkalemia [43]. In addition, other clinical studies have shown that the incidence of hyperkalemia decreased when the dose of eplerenone was reduced [51]. To ensure safety while reaping the benefits of reducing albuminuria, submaximal doses of MRA are suggested: <25 mg daily for spironolactone; <50 mg daily for eplerenone [52]. However, it is important to remember that as the dose is reduced, the beneficial effects of MRA therapy are also minimized.

Another important aspect is a diet low in potassium. All patients at high risk of hyperkalemia are recommended an intake of ≤2–3 g of potassium per day. The most important foods to watch out for are fruits such as strawberries, bananas, kiwi, and citrus fruits as well as vegetables such as beets, broccoli, carrots, corn, and cauliflower. In addition, energy drinks should be eliminated from the diet and the intake of meat, nuts, and cereals should be limited [53].

Other medications taken by the patient should also be looked at, as some of them may adversely affect K^+^ homeostasis (Table 5) [53,54].

In recent years, a new drug has appeared that has raised hope in the treatment of hyperkalemia associated with the use of drugs acting on the renin–angiotensin–aldosterone system in patients with chronic renal failure. Patiromer is a nonabsorbable cation exchange polymer containing a calcium sorbitol complex as a counterion. It works by binding the potassium released from food within the intestine, thereby lowering the serum potassium concentration [55]. Matthew R. Weir confirmed in his study that treatment with patiromer significantly reduces serum K^+^ and maintains normokalemia in patients ≥65 years of age and with CKD during therapy with RAAS blockers. Moreover, it reduces the risk of recurrent hyperkalemia [44].

## 11. Conclusions

Higher levels of proteinuria are a significant risk factor in the progression of kidney disease in patients with diabetes and non-DN. The above-mentioned studies have shown that MRA therapy causes a significant reduction in proteinuria, which results in a delay of progression in renal failure. In addition, the combination of MRA with ACEI/ARB results in a significant reduction in UAE and UACR levels compared to their values after only ACEI/ARB treatment and leads to a reduction of urine protein/albumin excretion.

The studies we have cited suggest that MRA treatment may be a beneficial therapy to reduce cardiovascular mortality in CKD patients. This is an important aspect as these diseases are the leading cause of death among ESRD patients. Spironolactone has been shown to be effective in reducing LVH and can be safely used [39], while currently, the drug with the greatest promise in reducing cardiovascular risk in patients with CKD is finerenone [40].

It is considered that eGFR and hyperkalemia may limit the widespread use of MRA in CKD patients. It should be emphasized that prior hyperkalemia was one of the exclusion criteria in most of the studies cited in our review. More detailed studies should be performed on people at an already elevated risk of hyperkalemia. Although most studies showed an increase in serum potassium after the introduction of MRA, the authors suggest that it could be controlled with a low-potassium diet, lower doses of MRA or cation exchange resins.

In summary, MRA may be one of the therapy options for patients with CKD, but the necessary management is regular monitoring of eGFR and potassium levels throughout the therapy.

## Figures and Tables

**Table 1:** The effects of MRA therapy on eGFR.

Authors	Year	Study Design	All Patients	Patient Category	Type of MRA	eGFR
Morales et al. [10]	2015	controlled clinical trial	71	patients with persistent proteinuria >1 g/24 h with any renal disease treated ACEI, ARB or their combination for more than 6 months	spironolactone	nonsignificant improvement
Kato et al. [11]	2015	multicenter, randomized clinical trial	52	patients with type 2 diabetes and diabetic nephropathy, aged 30 to 70 years, eGFR >30 mL/min/1.73 m^2^	spironolactone	decrease
Alexandrou et al. [12]	2019	meta-analysis	2767	patients with diabetic nephropathy, patients with nondiabetic proteinuric CKD, patients with resistant hypertension and diabetes mellitus, patients with hypertension and albuminuria	spironolactone, eplerenone, canrenone, finerenone	decrease
Bakris et al. [22]	2020	randomized, double-blind, placebo-controlled, multicenter clinical trial	5674	adult patients with type 2 diabetes and CKD treated with an ACEI or ARB at the maximum dose	finerenone	decrease
Ito et al. [24]	2020	multicenter, randomized, double-blind study	449	patients with type 2 diabetes and hypertension and UACR of 45 to <300 mg/g creatinine treated with a RAS inhibitor	esaxerenone	decrease
Minakuchi et al. [30]	2020	randomized controlled trial	141	patients with CKD stage 2 and 3 whose plasma aldosterone concentration was >15 ng/dL	eplerenone	remained at an equal level
Sun et al. [31]	2017	meta-analysis	1786	patients with type 2 diabetes treated ACEI/ARB alone or co-administration of MRA and ACEI/ARB	spironolactone, eplerenone, finerenone	did not improve

MRA—Mineralocorticoid receptor antagonists; eGFR—estimated glomerular filtration rate; ACEI—Angiotensin-converting-enzyme inhibitors; ARB—Angiotensin-II receptor blockers; CKD—Chronic kidney disease; UACR—Urinary albumin-creatinine ratio.

**Table 2:** The influence of combined MRA with ACEI/ARB therapy to proteinuria.

Authors	Year	Study Design	All Patients	Patient Category	Type of Treatment	Proteinuria Effect
Morales et al. [10]	2015	controlled clinical trial	71	patients with diabetes mellitus with or without obesity, with proteinuria levels >1 g/24 h	spironolactone/eplerenone with RAS blockers	proteinuria reduction
Kato et al. [11]	2015	multicenter randomized clinical trial	52	patients with type 2 diabetes and diabetic nephropathy, aged 30 to 70 years, GFR > 30 mL/min/1.73 m^2^ with albuminuria levels 100 mg/gCR–2000 mg/gCR	spironolactone with RAS blockers	UACR reduction
Ito et al. [24]	2020	multicenter randomized controlled trial	449	patients aged >20 years with type 2 diabetes, hypertension and UACR of 45 to <300 mg/g creatinine	esaxerenone with RAS inhibitors	UACR reduction
Wada et al. [25]	2020	multicenter randomized controlled trial	293	patients aged 20–75 years with type 2 diabetes	apararenone with/without ACEI/ARB	UACR reduction
Sun et al. [31]	2017	meta-analysis	1786	patients with type 2 diabetes	spironolactone/eplerenone/finerenone with ARB/ACEI	UAE/UACR levels reduction
Esteghamati et al. [32]	2013	randomized clinical trial	136	patients with diabetes mellitus and proteinuria UAE ≥ 30 mg/24 h	spironolactone with ARB/ACEI	UAE level reduction

RAS—Renin angiotensin system; UAE—Urinary albumin excretion.

**Table 3:** MRA effects on the cardiovascular system.

Authors	Year	Study Design	All Patients	Aim of the Study	Conclusion
Quach et al. [37]	2016	systematic review and meta-analysis	829	Assessment of safety and effectiveness of MRA in hemodialysis patients.	MRA may be identified as a profitable treatment reducing CV mortality in patients who require dialysis, but larger trials are needed.
Lin et al. [38]	2015	multicentric, randomized, placebo- controlled trial	235	Assessment of long-term effects and side effects of spironolactone in patients who require chronic dialysis.	Low dose of spironolactone may efficiently reduce the risk of death from CCV events in dialysis patients
Hammer et al. [43]	2019	randomized, placebo- controlled, parallel-group trial	97	Assessment the effect of spironolactone treatment on LVMI in patients who require hemodialysis.	Spironolactone treatment did not change LVMI significantly in comparison to placebo.
Feniman-De-Stefano et al. [39]	2015	randomized, double blind, placebo- controlled trial	17	Evaluation safety and effectiveness of spironolactone in reduction of the LVH in hemodialysis patients.	Spironolactone therapy was efficient and secure in the reduction of LVH in hemodialysis patients.
Filippatos et al. [40]	2020	randomized, double-blind, placebo- controlled trial	5674	Evaluation the effect of finerenone on CV and kidney function in patients with type 2 diabetes and CKD.	The use of finerenone lowers the overall risk of CV outcome in comparison to placebo. Treatment was profitable in patients with or without a history of CVD.

CV—Cardiovascular; CCV—Cardiocerebrovascular; LVMI—Left ventricular mass index; LVH—Left ventricular hypertrophy; CVD—Cardiovascular disease.

**Table 4:** Risk of hyperkalemia.

Authors	Year	Study Design	Type of MRA	Patient Category	Risk of Hyperkalemia
Alexandrou et al. [12]	2019	meta-analysis	spironolactone, eplerenone	9 study groups: patients with diabetic nephropathy 3 study groups: patients with nondiabetic proteinuric CKD 2 study groups: patients with resistant hypertension and diabetes mellitus 1 study group: patients with hypertension and albuminuria	no significant risk
Currie et al. [33]	2016	meta-analysis	spironolactone, eplerenone	patients with CKD stages 1–5 not requiring renal replacement therapy, with albuminuria or proteinuria	no significant risk
Lin et al. [38]	2015	randomized and placebo-controlled study	spironolactone	patients with ESRD undergoing stable hemodialysis from outpatient dialysis units or peritoneal dialysis	no significant risk
Feniman-De-Stefano et al. [39]	2015	double blind parallel RCT	spironolactone	patients on hemodialysis who presented with LVMI >51 g/m	no significant risk
Walsh et al. [45]	2015	parallel RCT	eplerenone	patients on hemodialysis	no significant risk
Itoh et al. [46]	2019	multicenter, single-arm, open-label	esaxerenone	hypertensive patients with type 2 diabetes and albuminuria	increased risk in patients with moderate renal impairment and type 2 diabetes and albuminuria

RCT, Randomized controlled trial.

**Table 5:** Medications that increase the risk of hyperkalemia.

Medication	Mechanism
nonsteroidal anti-inflammatory drugs	reduce renal blood flow, decrease renin secretion
trimethoprim	inhibits aldosterone-sensitive sodium channels in the distal nephron
cyclosporine	reduces potassium secretion in the collecting duct, interferes with renin release
azole antifungals	reduce aldosterone levels
heparin	inhibits aldosterone production
beta-blockers	reduce renin secretion and alter transmembrane potassium movement
angiotensin-converting enzyme inhibitors, angiotensin II receptor blockers	reduce potassium secretion

## Data Availability

The data used in this article are sourced from materials mentioned in the References section.
